# The role of short-term memory and visuo-spatial skills in numerical magnitude processing: Evidence from Turner syndrome

**DOI:** 10.1371/journal.pone.0171454

**Published:** 2017-02-21

**Authors:** Lucie Attout, Marie-Pascale Noël, Marie-Cécile Nassogne, Laurence Rousselle

**Affiliations:** 1 Psychological Sciences Research Institute, Catholic University of Louvain, Louvain-la-Neuve, Belgium; 2 Research Unit “Enfances”, University of Liège, Liège, Belgium; 3 Department of pediatric neurology, Saint-Luc University Hospital, Bruxelles, Belgium; Katholieke Universiteit Leuven, BELGIUM

## Abstract

Most studies on magnitude representation have focused on the visual modality with no possibility of disentangling the influence of visuo-spatial skills and short-term memory (STM) abilities on quantification processes. This study examines this issue in patients with Turner syndrome (TS), a genetic condition characterized by a specific cognitive profile frequently associating poor mathematical achievement, low spatial skills and reduced STM abilities. In order to identify the influence of visuo-spatial and STM processing on numerical magnitude abilities, twenty female participants with TS and twenty control female participants matched for verbal IQ and education level were administered a series of magnitude comparison tasks. The tasks differed on the nature of the magnitude to be processed (continuous, discrete and symbolic magnitude), on visuo-spatial processing requirement (no/high) and on STM demands (low in simultaneous presentation vs. high in sequential presentation). Our results showed a lower acuity when participants with TS compared the numerical magnitudes of stimuli presented sequentially (low visuo-spatial processing and high STM load: Dot sequence and Sound sequence) while no difference was observed in the numerical comparison of sets presented simultaneously. In addition, the group difference in sequential tasks disappeared when controlling for STM abilities. Finally, both groups demonstrated similar performance when comparing continuous or symbolic magnitude stimuli and they exhibited comparable subitizing abilities. These results highlight the importance of STM abilities in extracting numerosity through a sequential presentation and underline the importance of considering the impact of format presentation on magnitude judgments.

## Introduction

Numerous studies have emphasized the impact of two general factors in mathematical achievement: namely, visuo-spatial and short-term memory (STM) abilities.

### Impact of visuo-spatial abilities in number magnitude processing

First, processing geometric shapes, digits, mathematical symbols or even the numerosities of visual sets all require, to some extent, visuo-spatial skills (see [1] for a review). Some authors have proposed that the representation of number itself may be spatial in nature, with numerical magnitude representation organized along a mental number line (oriented left to right in Western culture) [2, 3]. In addition, various studies conducted in children as well as in adults have demonstrated the influence of perceptual variables which inevitably covary with numerosity (e.g. density, sum of perimeter, surface area, length, size,…) on performance in visual quantification tasks [4–7]. Some authors have even proposed that there is a unique system to represent all magnitudes, including number magnitude, space and time, each type of processing sustaining the development of the other. This is the case in the ATOM theory (a theory of magnitude) [8]. More precisely, some authors [8, 9] have proposed that numerical representation arises from repeated learning of the associations between spatial and temporal features. Thus, in this framework a deficit in spatial magnitude processing could disrupt the development of time and numerosity processing.

Dormal and Pesenti [6] assessed young adults’ ability to compare the numerosities or the lengths of two lines of dots in a Stroop paradigm. Both dimensions were manipulated to create congruent (the longer line had more dots), incongruent (the longer line had fewer dots) or neutral pairs (the relevant dimension varied while the other was kept constant). The authors reported that incongruent trials generally had a deleterious impact on performance, suggesting that the visuo-spatial (i.e., length) and the numerical dimensions had a mutual and bidirectional influence on each other. However, this reciprocal interaction seems to be asymmetrical depending on the presentation mode: the visuo-spatial dimension (length) had an impact on numerosity comparison with a simultaneous presentation [6] while the reverse pattern was observed with a sequential presentation [10]. A possible influence of working memory (WM) abilities in sequential presentation could explain these reverse profiles of results.

### Close links between visuo-spatial, STM and mathematical abilities

In addition to this relationship with visuo-spatial skills, the temporary retention in memory of information, whether visuo-spatial or verbal, also plays a crucial role in mathematical achievement (see [11] for a review). Longitudinal studies have demonstrated that STM and WM abilities assessed in preschoolers predict simple calculation abilities one and two years later during early childhood [12, 13]. Even if there is no direct evidence for this, the literature suggests an influence of STM/WM abilities on basic numerical processing. A well-known model of numerosity extraction, the accumulator model [14], proposed a preponderant role of WM in accumulating numerosity and in comparing this accumulation to numerical knowledge in long-term memory. On the other hand, some recent studies have suggested that WM ability has a major influence on number-space association. This number-space association is classically represented by the SNARC effect, corresponding to faster response for large numbers with the right hand than with the left hand and the reverse profile for the small numbers. Interestingly, Herrera, Macizo [15] observed a dissolution of the SNARC effect when the comparison task has to be performed under high WM load, demonstrating the role played by working memory in this number-space association. Moreover, van Dijck and Fias [16] showed that the SNARC effect was based on the position of numbers in STM rather than on their numerical size. Finally, neuroimaging studies have shown similar parietal activation for numerical, visuo-spatial and STM/WM processing [17–19].

In sum, these results underline the close relationship between WM/STM, visuo-spatial processing and numerical cognition and highlight the importance of considering these factors when assessing basic numerical processes. However, most studies on early magnitude representation have focused on the visual modality with no possibility of disentangling the influence of visuo-spatial skills and working memory (WM) abilities on visual quantification processes. Some current authors [20] have proposed that numerical representation could be independent of the modality of presentation, whether this is visuo-spatial or auditorily, sequential or simultaneous. In this study, we proposed exploring the impact of visuo-spatial and STM/WM deficit on basic numerical processes. According to Ansari [21], genetic syndromes are often highly relevant to understanding the origin of several developmental deficits and more specifically the developmental changes that give rise to failure in numerical cognition. In this respect, Turner syndrome is particularly interesting since it is characterized by a recurrent association of visuo-spatial deficit, weakness in WM abilities and mathematical learning disabilities, but with an IQ in the normal range.

### Using turner syndrome to explore this association

Turner syndrome is a genetic disorder resulting from the complete or partial loss of the second X chromosome present in females. The occurrence is approximately 1:2500 female births [22, 23]. The main physical phenotype is characterized by a short stature, a webbed neck, slowed and disproportionate growth, cardiovascular abnormalities, abnormal oestrogen production leading to delayed puberty or ovarian dysgenesis. Interestingly, at the cognitive level, most patients with TS exhibit a full scale intelligence quotient (IQ) within the average range [24]. Importantly, their verbal skills are essentially intact but they present visuo-perceptual disabilities which result in a clear discrepancy between verbal and non-verbal IQ [25–27]. Their visuo-perceptual deficits have been reported in a series of visuo-spatial processing tasks such as figure copy [28], drawing [29] and object localization. A single study also reported a purely visual processing impairment in a visual object recognition task [30] (but see [31], which reported normal-range performance in a form constancy task).

On the other hand, some authors [32] have proposed that visuo-spatial abilities such as mental rotation would be influenced by visuo-spatial WM capacities. Indeed, several authors have underlined a visuo-spatial short-term memory (STM) deficit in TS [33, 34]. Recent studies have demonstrated a link between poor performance in verbal and visuo-spatial N-back WM tasks and abnormal activation in the fronto-parietal network in TS [35–37]. From another theoretical perspective, Cornoldi, Marconi [38] carried out an in-depth exploration of visuo-spatial memory performance in four young adults with TS. The authors found a general deficit for visuo-spatial WM in several patients even though their profiles differed slightly in terms of visual and spatial WM components as well as for simultaneous and sequential STM presentations, suggesting some imprecision relative to their deficit. Finally, regardless of other cognitive functions, there is also some evidence of impairments in temporal processing for auditory stimuli, Turner patients being less efficient than controls in judging the difference between pairs of rhythmic patterns and pairs of tonal sequences but also in recognizing a famous melody once it had undergone a variation [39].

With regards to the numerical cognition domain, the risk of presenting mathematical learning disabilities is about four to five times greater among females with TS relative to the general population (prevalence of developmental dyscalculia is usually about 5 to 6%) (see [25, 28, 40, 41] for a recent review). Girls with TS struggle with math learning very early on, starting in kindergarten and persisting throughout primary and middle school [9, 28, 31, 41, 42] until adolescence [43, 44] and adulthood [45–47]. In the numerical domain, this population mainly have difficulties in arithmetic for applying procedures rather than for retrieving arithmetical facts stored in verbal memory, which is relatively well-preserved in this syndrome [45]. More specifically, previous data have revealed a consistent deficit in exact calculation, which is particularly clear in subtraction and in operations with large numbers requiring the deployment of procedures [45, 46, 48–50].

Regarding early quantification abilities, studies showed preserved verbal number sequence and counting skills in the TS population. However, results are inconsistent concerning symbolic numerical processing (assessed using digit comparison tasks and bisection tasks between two Arabic numbers) and subitizing abilities (numerical estimation tasks with flashed dots), relating to the ability to apprehend quickly and precisely a limited number of dots (4 approximately), with some studies reporting deficit while others do not. These differences are probably due to different matching criteria between TS and control participants in terms of IQ [9, 41, 45, 49]. With regards to non-numerical magnitude, only one study has assessed how girls with TS (mean age: 10 years 5 months) process continuous quantities using a length comparison task [9]. Patients with TS were slower and less accurate than the control group matched on chronological age, suggesting that they have a deficit in apprehending continuous non-numerical magnitudes. Nevertheless, comparing length also recruits visuo-spatial abilities, which are known to be weak in TS and the methodology does not allow for separation of these confounds in the interpretation of the deficit. In addition, patients with TS had significantly lower IQ abilities than their controls matched on chronological age (more than 20 points on global intellectual efficiency measures), leaving open the possibility that this confound might have contributed to the group difference. To our knowledge only one study has explored other non-symbolic number magnitude processing [41] using a collection comparison task from a standardized test (the TEMA-2) and they found no difference between children with TS and a control group of the same chronological age. To sum up, these studies suggest preserved verbal quantification processes (verbal sequence and counting) and numerical magnitude processing (collection comparison) while non-numerical magnitude processing (length comparison) was impaired.

### The present study

Accordingly, the aim of the present study is to conduct a systematic examination of basic quantitative processing in TS and to take into account the possible influence of visuo-spatial and WM/STM factors on these quantitative processes. More precisely, and in order to examine the various facets of magnitude representation, the tasks contrasted different kinds of magnitude processing, namely, continuous quantities (length and duration comparison tasks), discrete non-symbolic quantities (sound sequence, dot sequence, and visual sets of dots) and finally, symbolic magnitude (verbal and Arabic numerals). To specify the influence of visuo-spatial processing on numerical abilities, participants were presented with a series of magnitude comparison tasks contrasting different visuo-spatial processing demands (low vs. high visuo-spatial processing requirement). Obviously, the visuo-spatial skills involved in each kind of task are not necessarily the same. For instance, Arabic number comparison involves only the visual recognition of the symbols [51]. Length comparison should require low-level visuo-spatial processing, more related to perceptual processes and to the ventral stream, while the collection comparison task should involve higher-level visuo-spatial processes, closer to the dorsal stream and requiring the integration of several steps, from the perception of the dots and the normalization of these one to considering each dot as a numerical entity independently of these perceptual properties (see also [52]’s model for this view). Employing these different tasks allows us to explore several visuo-spatial requirements and different magnitude processing tasks, at a non-symbolic level for continuous and discrete processing and at a symbolic level.

Moreover, our numerical tasks varied as a function of STM load, the simultaneous presentation imposing a lesser load in STM than the sequential presentation, where the numerical representation has to be constructed item by item. In addition, we controlled the influence of WM by assessing the several components of WM and especially the visuo-spatial STM since several recent studies suggest that it is needed for the spatial coding of numbers to occur. Finally, we also assessed subitizing abilities, another crucial basic numerical processing task relying on visuo-spatial skills [53].

Therefore, our assumptions are as follows: if the visuo-spatial and STM/WM disabilities of TS participants impact their numerical processing, we expect a specific deficit in comparison tasks involving some visuo-spatial abilities such as length and collection comparison tasks as well as in tasks with higher STM demands, as is the case for the sequential presentation of dots/sounds comparison tasks and the subitizing task.

Alternatively, a deficit in all magnitude comparison tasks, whatever the visuo-spatial and the STM requirement, would be interpreted as a central deficit of basic magnitude processing according to the ATOM theory [8]. Indeed, according to this assumption, an original disability in visuo-spatial skills should lead to a numerical processing impairment observed whatever the modality of presentation, with or without visuo-spatial or STM requirement.

## Method

### Ethics statement

The experiment was conducted in accordance with the Declaration of Helsinki and the experimental protocol was approved by the regional ethical committee for biomedical research of the Department of Medicine of the Catholic University of Louvain which is in charge of the investigation in patients (Record number: B403201111579). All participants or their parents (for younger participants) gave a written consent.

### Participants

The experimental group consisted of twenty female participants with TS aged between 7 and 33 years (M = 18.5 years, SD = 89.1 months) recruited from the pediatric unit of a university hospital. There is no exclusionary criterion to recruit our Turner group excepted for the hearing loss and the uncorrected vision problems (needed to handle with our tasks). With regards to the medical history, 9 TS participants suffered from a heart surgery during childhood, 10 had no cardiovascular anomalies and no specific information about this has been found for one participant. All patients had received growth hormones during their childhood (excepted one child) and 14 had been treated with estrogen since the normal age of puberty. The diagnosis of the Turner syndrome was made by the study of the standard karyotype with at least 20 mitoses analyzed. In cases of complex mosaicism and in clinical situations in favor of a Turner syndrome with a normal standard karyotype, analysis of 100 mitoses was performed. Our population was composed of 12 participants with a monosomy 45XO and 8 with mosaic 45X karyotypes. Regarding these patients with mosaicism, the number of cells of each karyotype was unknown for only two of them. Among the others, three patients have a majority of cells (more than 75%) with 45XO, two patients have a karyotype with 45X/46XX/47XXX cells and one has the half of her cells with a 45XO karyotype. The Turner group was mainly composed of adolescents and adults (15–18 years: N = 5; more than 20 years: N = 9), and there were 6 children (from 7 to 12 years old). Twenty typically developing girls and women were individually matched to the TS group on verbal IQ (± 3 gap points of the raw score) and educational level for children or educational background for adults. We chose these two matching variables since verbal IQ was supposed to be at the same level in both groups, unlike visuo-spatial abilities, and the educational level allowed us to be sure that possible mathematical abilities differences were not due to a different learning level, some of them having repeated a grade. The control group was mainly composed of adolescents and adults (14–17 years: N = 5; more than 20 years: N = 9), and there were 6 children (from 6 to 12 years old). The control group had no history of neurological/psychiatric disorder, hearing impairment or learning disabilities and followed a standard school curriculum (without repeating a grade) as determined by their own or a parent’s report. Of the twenty participants with Turner syndrome, twelve reported mathematical difficulties at school and in everyday life. All adults had or were studying for a Bachelor’s degree while all children followed a general curriculum except one who was in vocational studies. In the Turner group, seven participants had repeated at least one grade (the 7 girls with TS who repeated a grade were 2 children and 5 adolescents from 15 to 18-years old). All participants were tested with four subtests from the Wechsler Intelligence Scale for Children-4th edition (WISC-IV; Wechsler, 2005) or the Wechsler Intelligence Scale for Adults-3rd edition (WAIS-III; Wechsler, 2000) depending on their age. There were administered two verbal subtests: the Vocabulary subtest assessing verbal knowledge and the Similarities subtest assessing verbal reasoning; and two non-verbal subtests: the Picture concepts subtest assessing the formation of non-verbal concepts and their categorization, and the Block design subtest assessing visuo-spatial construction.

### Material

#### Mathematical fluency tasks

Four arithmetic fluency tasks were administered. The first three tasks were *single-digit arithmetic fluencies* involving, respectively, additions, subtractions and multiplications. For each operation, participants had 150 seconds to solve as many problems as possible (written response). Addition and multiplication problems were drawn from all possible combinations of the integers 1–9 and the set of subtractions was the exact counterpart of the addition set. These combinations resulted in a total set of 81 problems for each operation, respectively. The last task examined *complex arithmetic fluencies* and consisted of intermixed complex addition, subtraction and multiplication problems. Participants had 150 seconds to solve as many problems as possible (written response). Twelve problems were presented for each operation, resulting in a total set of 36 complex problems. Out of these, four problems in each operation could be solved using short-cut strategies (e.g. 45 + 99 = 45 + 100–1 or 13 × 5 = (13 × 10) / 2). All additions and subtractions comprised at least one two-digit number and required carrying (additions) or borrowing (subtractions). Multiplication problems were composed of one single-digit and one two-digit operand. For each of these four fluency tests, the experimenter scored the number of correct responses given in the allocated time and the number of errors.

#### Working memory tasks

The three main components of WM defined in Baddeley and Hitch’s model [54, 55], namely, the phonological loop, the visuo-spatial sketchpad and the central executive component, were individually examined in tasks that did not require the recall or manipulation of numerical content. These tasks were the same as those used in Rousselle, Dembour [56]. Phonological loop capacity was assessed in a forward letter span task (3 to 9 letters). Sequences of letters (starting with 2) were presented orally to the participant at a rate of one per second and the participant had to repeat the letter in the same order. Sequences increased in length. The VSSP was assessed with a two-dimensional visuo-spatial span task inspired from the Corsi task including a span from 2 to 10 and where participants had to remember the location of a series of cells in a blank grid previously touched one by one by the examiner, at the rate of one cell per second. The participant had to place tokens on the cells previously touched by the examiner (2 tokens were offered for span 2, 3 for span 3 and so on). Finally, the central executive component was assessed with a category-span task. One-syllable words were presented orally at the rate of one word per second to the participant who had to repeat them after starting with the food words and then the animal words. Sequences started with 2 words, reaching a maximum of 9 words. For all tasks, participants had to succeed in two trials of the same difficulty (or number of items) to access a higher level (span +1). The tasks ended when a participant failed at two (or more) out of the three trials for a given difficulty level. Each correct response was credited with one point. The dependent variables correspond to the total of points credited.

#### Magnitude comparison tasks

**Fig 1:**
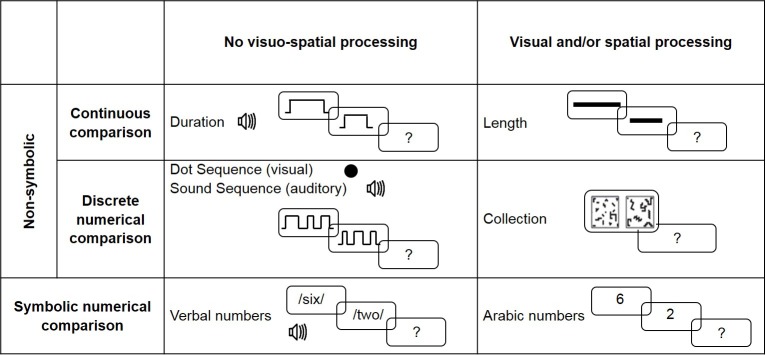
Description of the magnitude comparison tasks.

Seven magnitude comparison tasks were used (see Fig 1). There were five non-symbolic magnitude comparison tasks. These tasks differed in the nature of the magnitudes to be compared, in the emphasis put on visual and/or spatial and STM processing. There were two continuous magnitude comparisons (length comparison and duration comparison) and three discrete numerical comparison tasks using different presentation modes (collection comparison, dot sequence comparison and sound sequence comparison). The two other tasks assessed symbolic numerical magnitude processing through verbal and Arabic numerical comparisons.

Each of the tasks was carried out on a tablet PC (HP Elitebook 2740p, Screen: 12.1-inch WXGA (1280x800)). Stimuli were presented on a navy blue background with the E-Prime experimental software (Version 1.1, Psychology Software Tools, Inc., Pittsburgh, PA). Participants had to choose between two possible responses, one presented on the left and the other on the right side of the screen, by touching the screen with the tactile pen on the side of the correct response. The tactile screen surface was divided by an invisible vertical midline defining two equal response zones. Instructions emphasized both speed and accuracy. They were repeated as often as necessary to keep participants on task.

In the five non-symbolic magnitude comparison tasks, the difference between the quantities to be compared varied along six different ratios: 1/2, 2/3, 3/4, 5/6, 7/8, 8/9. Two different pairs of magnitudes were presented by ratio. Table 1 presents the pairs of numerosities which were used in the discrete numerical comparison tasks for each ratio. These ratios of increasing complexity were introduced progressively throughout the task to determine individual sensitivity to magnitude difference in each task. Participants always started with stimuli pairs varying according to the two easiest ratios, that is, 1/2 and 2/3. Fewer and fewer discriminable ratios were then progressively introduced (3/4, 5/6, 7/8, and finally 8/9), depending on the participant’s correct response rate for each ratio. Pairs of consecutive ratios were always intermixed with each other so that stimulus pairs of one ratio were never presented alone. The task was discontinued when a participant performed at chance level for two out of three consecutive ratios. This procedure was adopted to take into account the participant’s individual limits regarding their sensitivity to magnitude differences but also their own attentional capacities. Indeed, presenting participants with so many ratios that they are not able to discriminate could be discouraging and could lead them to adopt ‘‘guessing” strategies [57] that would have introduced a lot a noise into the data, including on easy ratios that could be in fact be well discriminated.

**Table 1:** Pairs of magnitudes presented in discrete numerical comparison tasks.

Ratios						
	1/2	2/3	3/4	5/6	7/8	8/9
**Small**	7–14	6–9	6–8	5–6	7–8	8–9
**Large**	8–16	10–15	12–16	10–12	14–16	16–18

In each task, the side of the correct response was counterbalanced: each pair appeared four times, twice with the larger magnitude on the right side and twice with the larger magnitude on the left side. When all ratios were presented, participants were administered a total of 48 stimulus pairs in each task (2 pairs x 2 sides x 2 presentations x 6 ratios). Throughout the experiment, pairs were presented in a pseudo-random order (i.e. no identical pairs in two consecutive trials, no more than three consecutive correct responses on the same side and no more than two identical ratios in succession). Before beginning each task, participants performed six practice trials with pairs of magnitudes differing by a 1/3 ratio in order to check the understanding of the instructions.

Continuous magnitude comparison tasks. These tasks involved no numerical processing and required participants to process the duration or the length of continuous stimuli respectively presented in the auditory (no visuo-spatial processing) or in the visual modality (visuo-spatial processing requirement). They come from Rousselle, Dembour [56]. In the *Duration Comparison task*, participants had to compare the duration of two identical sounds presented successively while in the Length Comparison task participants had to compare the length of two white lines (see more details in [56]). In order to equilibrate the memory load of the two continuous magnitude comparison tasks, the lines were presented one after the other, as was also the case with the sounds presented in the duration comparison task.

Discrete numerical comparison tasks. The three non-symbolic numerical comparison tasks assessed participants’ ability to process the discrete numerical properties of sets presented either sequentially (Dot Sequence or Sound Sequence, low visuo-spatial requirement and high WM demands) or simultaneously (Collection, high visuo-spatial processing requirement and low WM demands). In the Dot Sequence Comparison task, participants had to compare the numerosities of two sequences of flashed dots presented in succession. The stimulus was a single white dot (diameter: 3.5 cm) flashed rapidly in a single location on the left and then on the right side of the screen. To prevent participants from basing their judgment on perceptual non-numerical dimensions, the sequences were constructed using non-periodic signals to avoid rhythm and pattern recognition (for more details, see [58, 59, 60]). Moreover, numerosity and cumulated duration were manipulated in two congruity conditions. In congruent trials, the larger sequence in number was also the longer in duration while in incongruent trials, the larger sequence in number was the shorter in duration. The inter-stimuli interval (ISI) duration and the dot presentation time (D) were cumulated to obtain the total duration. The total duration varied from 1500 ms to 4500 ms while the ISI and D durations varied from 66.7 to 300 ms. The shortest and the longest ISI and D duration were the same within the sequences to be compared (smallest: 66.7 ms, longest: at least 200 ms). The trial started with the presentation of two red fixation crosses displayed respectively on the left and right side of the screen. When the participant was judged to be visually attending to the display, the experimenter triggered the disappearance of the left cross followed by the presentation of the first sequence, on the left. Then, the left fixation cross reappeared and the right cross disappeared before the presentation of the second sequence on the right side of the screen. Participants had to select the sequence with the larger number by touching the side of the screen where the most dots were presented. Instructions emphasized that the duration of the sequence was not important. Participants were instructed not to count the flashed dots as they would not have the time to do so. They could respond as soon as they got the answer, with no time limit after stimuli disappearance. In the Sound Sequence Comparison task, participants had to compare the numerosities of two series of tones presented in rapid succession. The task was constructed in exactly the same way as the dot sequence comparison but dots were replaced by identical tones (audio format: 44100 Hz, 32 bits, Mono). To attribute a location to the played sounds, two ears were displayed on the left and right side of the screen respectively throughout the task (black and white drawings of ears covering both 18.7° of visual angle). The trial was initiated by the experimenter when the participant was judged to be visually attending to the display. The left ear was first surrounded by a red frame for 700 ms. Three hundred milliseconds after the disappearance of the left red frame, the first sequence was diffused bilaterally by the computer speakers. After a variable delay (between 150 and 1125 ms), a red frame was displayed for 700 ms around the right ear. The second sequence was then diffused 300 ms later. As for the Dot Sequence comparison, participants had to select the sequence with the larger number by touching the side of the screen with the ear that ‘‘heard” the larger number of sounds. They could give a response from the beginning of the second sequence with no time limit. In the Collection Comparison task, participants were asked to compare the numerosities of two collections displayed simultaneously on the screen. Stimuli consisted of two white boxes containing black pieces of puzzle. In order to control as much as possible the influence of perceptual non-numerical dimensions on the participants’ judgment, the numerosity and the cumulated black area were manipulated and counterbalanced between both conditions, congruent and incongruent (see in [56] for more details).

#### Symbolic numerical comparison tasks

Participants’ abilities to process the numerical magnitude conveyed by symbols were assessed using Verbal and Arabic Number Comparison tasks which requested participants to compare the magnitude of verbal vs Arabic numerals, respectively (see Fig 1). The verbal task required no visuo-spatial processing while the Arabic Number Comparison involved the processing of the visual form of the numbers.

Both tasks followed exactly the same procedure and participants were invited to select the larger of two numerals presented successively (Arabic numbers: Arial, 48-point font; Verbal numbers: 44100 Hz, 32 bits, Mono). The stimuli varying along the ratio comprise the same stimuli as in the non-symbolic comparison tasks (see Table 1). In both tasks, the side of the correct response was counterbalanced: each pair appeared four times, twice in ascending (e.g. 2–3) and twice in descending (e.g. 3–2) order. We also included 40 unanalyzed filler pairs (e.g. 1–2) in order to ensure that all numerals were considered equally as the smaller and the larger of a pair. Hence, we obtained a total of 92 pairs (52 pairs for the ratio set and 40 pairs for fillers). Both tasks started with the presentation of small pairs (i.e. including only numbers smaller than 10). The second part of the task with larger numbers (from 10 to 32) was administered only if the participant had reached a criterion of more than 50% accuracy. For data analyses, we considered median correct response times (RT) in function of ratios.

#### Subitizing task

Participants were briefly presented with arrays of 1 to 7 dots and were asked to say out loud ‘how many’ dots were presented as quickly and accurately as they could. Stimulus presentation and response recording were carried out on a tablet PC (HP Elitebook 2740p, Screen: 12.1-inch WXGA (1280x800)) using both a voice key (latency recording) and a numerical pad (accuracy measurement). Stimuli were presented on a grey background with the E-Prime experimental software (Version 1.1, Psychology Software Tools, Inc., Pittsburgh, PA). Each trial started with the presentation of a central red fixation cross for 500 ms, followed by the display of the target collection of 1 to 7 dots for 200 ms. The collection was then immediately occulted by a mask for 500 ms. Finally, a screen with a question mark was presented until participants gave their response orally. The verbal response triggered a voice key (latencies) and the experimenter then recorded the participant’s response on a numerical pad (accuracy). The stimuli consisted of 1 to 7 randomly arranged black dots of equal size (6mm in diameter), plotted randomly in the cells of a 6x6 virtual matrix, comprising the same 5.3x5.3x8 area as the premask. Each numerosity was presented six times in different configurations. By contrast, the mask consisted of dots of heterogeneous size and covered the whole surface of the screen consisting of dots. The experiment started with seven practice trials.

#### Processing speed assessment

A stimulus detection task was used as a measurement of general processing speed to examine whether processing speed differences might account for participants’ performance in the magnitude comparison tasks. A white dot appeared on the left or right side of the screen and participants were asked to touch the dot with the tactile pen as fast as possible. This task provides a reliable measure of the time necessary for stimulus detection and response production.

#### Speeded counting task

The aim of this task was to determine participants’ counting speed, in order to be able to appreciate their ability to use counting in the collection comparison task. Participants were presented with eight strings of 6, 7, 8, 9, 12, 14, 16 and 18 dots in a fixed pseudo random order and were asked to count them as fast as they could and to tell how many dots were displayed. Dots were arranged linearly to facilitate the distinction between the dots counted and those yet to be counted. Participants were specifically asked to count dots one by one. The counting speed was assessed as the average counting time by item calculated over all correct trials (i.e. total counting time divided by the number of dots to be counted for each correct trial).

### Experimental procedure

Participants were tested individually in a quiet room. Testing was completed in two 75 minutes sessions, approximately, depending on participants’ performance and attentional level. The first session started with the four IQ subtests (the Block Design, the Similarity, the Picture Concepts and then, the Vocabulary subtest) followed by the three WM subtests. The tasks assessing arithmetic opened the second session and were followed by computerized basic numerical comparison tasks.

## Results

### Descriptive measures

Table 2 reports descriptive information regarding age, IQ, WM measures, and arithmetical fluencies in the TS and C groups. As control participants were matched on verbal intelligence raw score with their TS participants (± 3 gap points), the two groups did not differ in the two verbal intelligence subtests. Only one participant with TS was not perfectly matched with his control on verbal intelligence (7 gap points). This TS child exhibited a verbal intelligence level of kindergarten, lower than his current grade level, but succeeded in symbolic comparison tasks and mathematical fluency tasks, measures more dependent to formal school learning taught in primary school. Thus, if we had really wanted to match a control child at verbal intelligence level, we would have compared the TS participant to his control only on the non-symbolic comparison tasks. Paired-samples t-tests revealed no significant group effect on the Picture concepts subtest but a significant difference on the Block design subtest, with lower performance for the TS group (see Table 2). The performance in this subtest confirms the presence of a visuo-spatial impairment in the TS group, as usually reported in the literature [61]. Finally, the TS group also showed weaker capacities than the C group for both STM measures (visuo-spatial sketchpad and phonological loop), but not for the verbal central executive component of WM.

**Table 2:** Data and paired t-tests for general measures in TS and C groups.

	TS group		C group			
	Mean	SD	Mean	SD	t	p
**Age (months)**	219.20	87.09	219.75	91.75	-.21	.83
**IQ measures**						
*Vocabulary* (max. 68)	32.85	11.08	33.90	10.21	-1.57	.13
*Similarities* (max. 44)	20.15	6.12	20.35	6.11	-.45	.66
*Block design* (max. 68)	35.40	11.50	42.45	10.07	-3.45	**.003**
*Picture concepts* (max. 28)	17.45	4.32	18.70	2.92	-1.70	.11
**Working memory**						
*Visuo-spatial sketchpad* (max. 42)	35.15	7.00	38.75	5.54	-2.52	**.02**
*Phonological loop* (max. 16)	7.70	1.63	9.00	2.29	-2.80	**.01**
*Central executive* (max. 16)	6.75	1.86	7.25	2.20	-.85	.41
**Mathematical fluency**						
*Addition (Accuracy)* (max. 81)	42.55	23.51	49.95	23.53	-1.90	.07
*Subtraction (Accuracy)* (max. 81)	33.75	20.19	40.00	18.32	-2.01	.06
*Multiplication (Accuracy)* (max. 81)	25.05	17.18	34.50	16.21	-2.74	**.01**
*Complex arithmetic (Accuracy)* (max. 36)	10.71	5.02	13.65	5.29	-2.53	**.02**
**Counting speed (ms/item)**	437.83	151.26	433.20	113.70	.14	.89
**Speed processing (ms)**	567.62	98.11	565.40	95.28	.08	.94

Given the heterogeneity of mathematical achievement levels in our samples, the complex calculation task could not be administered to the younger participants (thus, N = 17). We observed marginally significant difference between both groups for addition and subtraction fluencies (Ps < .07) while the two groups differed on multiplication fluency and complex calculation (Ps < .02). Finally, there were no significant differences on the speed processing task or counting speed between both groups, as we can see in Table 2.

### Non-symbolic magnitude comparison tasks

Statistical analyses were carried out on the Weber fraction (w). This was estimated individually on the basis of participants’ correct response rates in each ratio and each task in order to assess the precision of the underlying non-symbolic magnitude representations. The Weber fraction computation was based on Pica, Lemer [62] and Halberda and Feigenson [57] and used the algorithm developed by Rousselle, Dembour [56]. The Weber fraction is a reliable index of participants’ sensitivity to magnitude difference and reflects the variation of participants’ performance as a function of the ratio between the magnitudes to be compared. This measure was preferred rather than accuracy since all participants did not necessarily achieve the same amount of items. Given the disparity in the participants’ age in both groups, chronological age was entered as a covariate in all analyses.

For the continuous magnitude comparison tasks, a repeated measures ANCOVA with Task (Duration vs. Length) as within-subject factor and Group (TS vs. C) as between-subjects factor was run on the Weber fractions, with age as a covariate. This analysis showed a significant effect of task (F(1,37) = 8.88, η^2^ = .19, p < .01) with a higher sensitivity to magnitude differences in length than in duration comparison but no difference between groups (F(1,37) = .38, η^2^ = .01, p = .54) and no interaction (F(1,37) = .72, η^2^ = .02, p = .40).

For the discrete magnitude comparison, an ANCOVA on discrete magnitude tasks with Task (Dot Sequence vs. Sound Sequence vs. Collection) as within-subject factor and Group (TS vs. C) as between subject factor was conducted on the Weber fractions, with age as covariate. This analysis showed no task effect (F(2,74) = .91, η^2^ = .02, p = .41) but a significant difference between groups (F(1,37) = 8.71, η^2^ = .19, p < .01): indicating that participants with TS reached a lower level of precision than the C group. Importantly, the group by task interaction was also significant (F(2,74) = 3.25, η^2^ = .08, p < .05). Planned comparisons showed that the numerical acuity of the TS group was reduced in the dot sequence (F(1,37) = 4.37, η^2^ = .11, p < .05) and sound sequence comparison tasks (F(1,37) = 8.16, η^2^ = .18, p < .01) compared to the C group. By contrast, the numerical acuity of the two groups did not differ in the collection comparison task (F(1,37) = .58, η^2^ = .02, p = .45).

**Fig 2:**
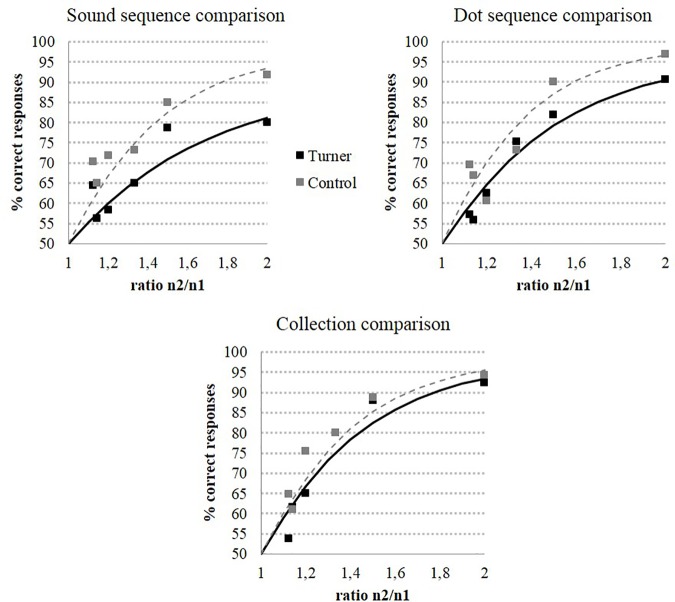
Accuracy data by group for discrete magnitude comparison tasks as a function of ratio.

**Fig 3:**
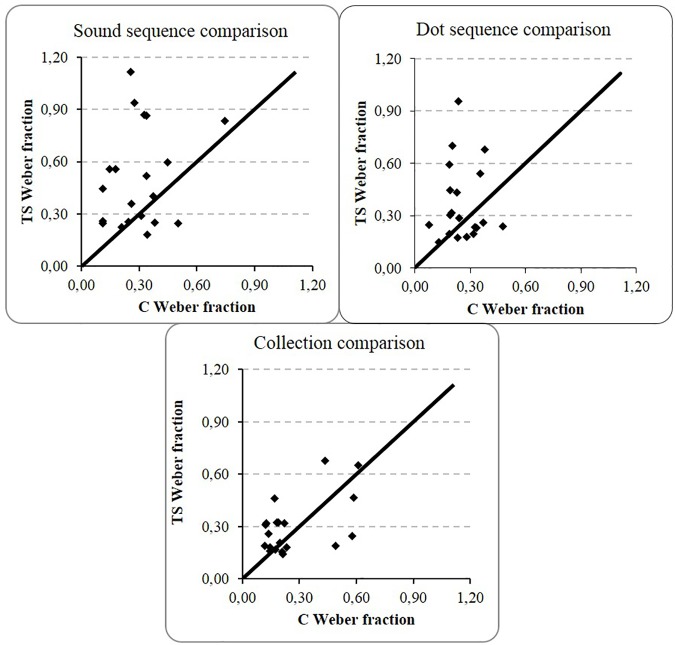
Association between Weber fractions within each Turner Syndrome (TS)—Control (C) pair for discrete magnitude comparison tasks. The central line represents the perfect match.

Fig 2 represents the percentage of correct responses according to the ratio of the pairs in each task for both groups while Fig 3 illustrates the distribution of the Weber fractions for each TS-C pair of participants in discrete numerical processing tasks. The central midline represents the hypothetical situation of a perfect correspondence in all pairs of participants between the Weber fractions of the TS and the matched C participants. As can be seen for the two sequential tasks, the majority of dots is distributed above this line which indicates a higher and therefore a less precise representation in the TS group.

Overall, TS participants exhibited a specific impairment in number magnitude representation only when numerosities were presented sequentially, one element at a time, and this for both auditory and visual presentation formats.

### Symbolic magnitude comparison tasks

The two groups performed near ceiling in both symbolic numerical comparison tasks (Arabic numbers: TS: mean = 96.67%, SD = 4.99; C: mean = 97.92%, SD = 3.45; verbal numbers: TS: mean = 97.50%, SD = 5.47; C: mean = 98.54%, SD = 3.39). Accordingly, RTs were adjusted to reflect both speed and accuracy using the formula RT/(1 –error rate) (see [9] for a similar method. A 6 × 2 × 2 repeated measures ANCOVA with tasks (Arabic vs. verbal) and ratios (1/2 vs. 2/3 vs. 3/4 vs. 5/6 vs. 7/8 vs. 8/9) as within-subject factors and group (TS vs. C group) as a between-subjects factor, with age as the covariate, showed a significant main effect of ratio (F(1,36) = 2.84, η^2^ = .07, p < .05) but no main effect of task, group or any interaction (all Ps > .05). Thus, both groups performed similarly when processing the magnitude of symbolic numbers whether they were presented in the auditory or the visual modality.

### Subitizing

Accuracy data were analyzed in a 7 × 2 repeated measures ANCOVA controlling for age with numerosity (1 to 7) as a within-subject factor and group (TS *vs* C group) as a between-subjects factor. This analysis yielded a main effect of numerosity (F(6,216) = 9.27, η^2^ = .20, p < .001) but no difference between groups (F(1,36) = 1.05, η^2^ = .03, p = .31) and no interaction effect (F(6,216) = .25, η^2^ = .00, p = .96). A Newman-Keuls post-hoc test on quantity demonstrated that the three smallest numerosities did not differ from each other (from 1 to 3, all Ps>.05) but all of them differed significantly from the four largest ones (from 4 to 7, all Ps < .05). All numerosities above 4 differed from each other (Ps < .05). The data followed a quadratic trend (F(3,111) = 4.62, η^2^ = .11, p < .01) characterized by a steep decline in performance starting at 4. The absence of interaction indicates that both groups showed a similar precision to apprehend quickly numerosities smaller than 4 (corresponding to the subitizing range) but also to estimate a larger quantity of dots. For RTs, we computed the mean between two successive numerosities (1–2; 3–4; 5–6) in order to have sufficient RTs to calculate a valid measure. A 3 × 2 repeated measures ANCOVA controlling for age with range of numerosity (1–2; 3–4; 5–6) as a within-subject factor and group (TS *vs* C group) as a between-subjects factor was conducted. This analysis yielded a main effect of the range of numerosity (F(2,72) = 17.63, η^2^ = .33, p < .001) but no difference between groups (F(1,36) = 1.11, η^2^ = .03, p = .30) and no interaction effect (F(2,72) = .60, η^2^ = .02, p = .55). A Newman-Keuls post-hoc test on range of numerosity demonstrated that all ranges of numerosities differed significantly from each other (all Ps < .05).

### Role of counting speed, short-term and working memory

Additional analyses were run to better understand why the numerosity comparison was impaired only when dots or sounds were presented sequentially. This analysis was based on the premise that when we process numerosities presented sequentially, the magnitude information is not accessible all at once but has to be constructed progressively, by accumulating the elements over time. Accordingly, the to-be-constructed magnitude representation has to be stored and updated in STM in a kind of internal counter (see the *accumulator* metaphor [14]). Thus, STM and WM components could be specifically recruited in these tasks. Moreover, even if the frequency of events in the sequence (dots or sounds) was designed to avoid counting processing, we also examined whether counting speed had contributed to the performance in the two tasks requiring a sequential processing of numerosities. One way to address this issue is to determine whether performance in both tasks is still explained by group membership after controlling for verbal and visuo-spatial STM, verbal WM and counting speed. First, we used a partial correlation analysis to observe what measures could explain performance in sequential tasks, after controlling for the age effect. Neither of these two sequential tasks correlated with counting speed (dot sequence comparison: r_(38)_ = .02, p = .88; sound sequence comparison: r_(38)_ = .22, p = .18), confirming that this processing was not involved in the apprehension of these fast sequential numerosities. Weber fraction of the dot sequence comparison task was somewhat related with the two STM measures but none of these correlations reached statistical significance (VSSP STM: r_(38)_ = -.26, p = .12; Verbal STM: r_(38)_ = -.24, p = .14; Verbal WM: r_(38)_ = -.01, p = .93). By contrast, all of the correlations with the Weber fraction measured in the sound sequence task were significant (VSSP STM: r_(38)_ = -.49, p < .001; Verbal STM: r_(38)_ = -.43, p < .01; Verbal WM: r_(38)_ = -.35, p < .05). In a hierarchical regression analysis (see Table 3), we first entered age, and then visuo-spatial, verbal STM tasks and verbal WM task successively. Finally, we entered the group membership and we observed that this variable no longer explained performance in the dot or sound sequence comparison tasks after controlling previously for variance explained by STM and WM measures. These results suggest that differences between groups in sequential numerical tasks were mostly due to the difference observed in STM tasks, and this for both kinds of tasks.

**Table 3:** Hierarchical multiple regression analyses of group variables on Dot and Sound sequence comparison tasks, after controlling for age and WM/STM measures.

Measures	ΔR^2^	B	SEB	*Β*	*t(38)*	*p*
**Dependent variable**	**Dot sequence comparison (w)**					
1. Age	.08	-.00	.00	-.28	-1.80	.08
2. Verbal WM	.00	-.00	.02	-.02	-.09	.93
3. Visuo-spatial STM	.07	-.01	.00	-.27	-1.61	.12
4. Verbal STM	.06	-.02	.02	-.22	-1.13	.27
5. Group	.03	-.08	.06	-.23	-1.36	.18
**Dependent variable**	**Sound sequence comparison (w)**					
1. Age	.05	-.00	.00	-.22	-1.41	.17
2. Verbal WM	.12	-.05	.02	-.41	-2.27	**.03**
3. Visuo-spatial STM	.15	-.02	.01	-.45	-2.83	**.00**
4. Verbal STM	.05	-.04	.03	-.34	-1.70	.10
5. Group	.04	-.12	.08	-.23	-1.56	.13

### Number magnitude representation and arithmetic performance

**Fig 4:**
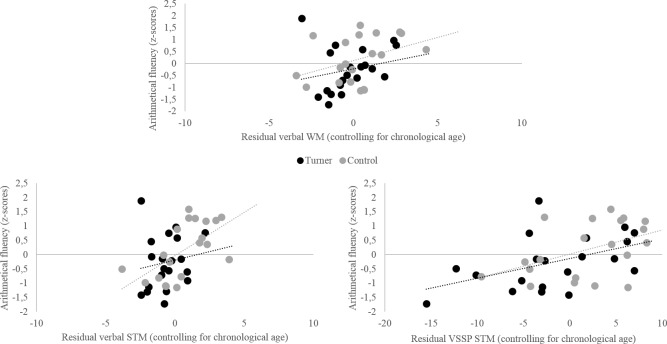
Partial correlations controlling for age between Arithmetic fluency scores and short-term and working memory scores for all participants, the TS and the C groups.

Finally, the relationship between number magnitude representation and mathematical achievement was examined in order to address the question of the origin of individual differences in mathematical achievement relative to basic numerical processes. This question was addressed using the sample as a whole, the TS group serving mostly to provide variability on the variable of interest. To obtain one measure of mathematical achievement, z-scores in each arithmetical fluency task were averaged in a single arithmetic score. Partial correlation analysis controlling for age was carried out to examine whether this arithmetic score correlated with verbal WM and STM capacities and performance in basic magnitudes comparison tasks (i.e. Weber fractions in non-symbolic comparison, adjusted RTs in symbolic comparison and the subitizing range). The subitizing range was estimated as the larger number size with performance reaching at least 83% (5 correct answers among 6 items by numerosity). Results indicated that arithmetic score correlated significantly with both STM abilities, verbal (r_(38)_ = .57, p < .001) and visuo-spatial (r_(38)_ = .59, p < .001), but also with verbal WM (r_(38)_ = .40, p < .05) (see Fig 4). With regards to the magnitude comparison tasks (high score in w or in adjusted RTs witnessing a poor performance), arithmetic score correlated significantly with symbolic comparison tasks (Arabic numbers: r_(38)_ = -.32, p < .05; verbal numbers: r_(38)_ = -.50, p < .01), with some non-symbolic comparison tasks (duration: r_(38)_ = -.34, p < .05; sound sequence: r_(38)_ = -.60, p < .001) and marginally with the subitizing range (r_(38)_ = .30, p = .06). However, the arithmetic score did not correlate with length (r_(38)_ = -.24, p = .14), collection (r_(38)_ = -.19, p = .24) and dot sequence (r_(38)_ = -.27, p = .10) comparison tasks.

Multiple stepwise linear regression analyses were then conducted on the mathematical achievement score, in the two groups together, to examine the unique contributions of these numerical (i.e. Weber fractions in non-symbolic comparison, adjusted RTs in symbolic comparison and the subitizing range) and memory (i.e. WM and STM) measures. Age was included in the model in order to account for the age difference between participants. The regression analysis produced a significant model (R = .81, adjusted R^2^ = .64; F(1, 37) = 35.74, η^2^ = .66, p<001), with both STM measures as the only significant predictors.

## Discussion

The aim of this study was to examine the relationship between visuo-spatial and WM/STM processes on basic quantitative processing. This issue was addressed in people with TS whose cognitive phenotype is characterized by a recurrent association of visuo-spatial, WM/STM deficits and mathematical learning disabilities. Seven magnitude comparison tasks bearing on different kinds of magnitudes (continuous, discrete numerical magnitudes and symbolic magnitudes) and putting different emphasis on visual-spatial processing (no visuo-spatial processing vs. visual and/or spatial processing demands) and on STM load (simultaneous vs. sequential presentation) were contrasted to assess the influence of visuo-spatial and WM/STM skills on magnitude processing.

### Summary of the results

First of all, the cognitive profile of our sample of patients with TS conformed to the cognitive phenotype described in the literature. Compared to control participants matched on verbal IQ and education level, people with TS presented specific deficits of visuo-spatial and STM abilities as well as an impairment in arithmetic fluency (especially for multiplication and complex calculation). However, the verbal component of WM seems to be preserved.

With regards to basic magnitude processing, the participants with TS presented similar abilities to compare continuous stimuli and this for both, visual and verbal presentations (i.e., length and duration comparisons). Importantly, for the non-symbolic numerical tasks, a dissociation was observed between the simultaneous and the sequential presentation. Participants with TS exhibited an impairment in the numerical comparison of a sequence of visual or auditory stimuli but similar performance with the C participants in comparing dot collections presented simultaneously, a task implying more visuo-spatial skills but low STM requirement. In symbolic magnitude comparison tasks, TS participants performed as fast and as precisely as the C group with both Arabic and verbal numbers. Finally, participants with TS did not show difficulty in enumerating small sets of elements very quickly and accurately.

### Influence of visuo-spatial difficulties in Turner syndrome’s mathematical disabilities

Overall our results in the TS sample do not support the visuo-spatial hypothesis stating that visuo-spatial difficulties could influence numerical acuity assessed by tasks with visuo-spatial demands. Indeed, we did not observe difference between the two groups for tasks involving higher visuo-spatial processes, namely the length and dot collection comparison tasks, nor for the subitizing task. A previous study showed an impairment in explicit length comparison abilities [9] in TS participants but the authors used two groups differing significantly in terms of IQ (gap of 25 points on Full scale IQ between the Turner and control group). Even if our TS participants demonstrated a significant difference on visuo-spatial skills (Block design subtest), they were matched on verbal IQ to the C group. In other words, the difference between groups observed for the length comparison task in Simon et al.’s study might be explained by the IQ confound in their study. Moreover, other tasks also involving visuo-spatial abilities, but to a lesser extent, such as subitizing apprehension and Arabic numerical magnitude comparison, were performed similarly by both groups. Therefore, in the TS group, the weakness in visuo-spatial abilities (see [61] for a review, and [63] for difficulties in figure reproduction) did not seem to affect basic numerical processes.

Several authors have speculated about a possible global magnitude deficit in people with TS [45] but our results argue against this hypothesis. In fact, very few data have been reported in the literature to support the hypothesis of a global magnitude processing deficit in this syndrome (see for a review [40]). Indeed, patients with TS exhibited no difficulty in counting, enumeration, number magnitude judgments and number line bisection tasks [9, 41, 45]. Subitizing abilities, considered as one of the foundations of numerical cognition development, have been found to be impaired in people with TS in previous studies with adults [45] and children [9]. In our study, with a group mainly composed of adolescent and adults, there was no difference in the subitizing range, and hence we did not replicate the finding of Bruandet et al. [45]. However, the methodology used was quite different. We presented the collections for a very short duration (200 ms) followed by a mask to clearly prevent any attempt to count, in contrast to the studies of Bruandet et al. (45) and Simon et al. [42] where the stimulus presentation time was unlimited. Finally, as already underlined, Simon’s study used a control group matched with the TS group on chronological age only so that the full scale of IQ was clearly confounded with the group and hence did not allow an understanding of the origin of the difficulties. We believe that a match on verbal IQ but also on educational level provided better conditions to compare these two groups on magnitude representation. Moreover, the ATOM theory was not completely supported by our results or at least the deficit did not appear at the general magnitude level. Indeed, a deficit in visuo-spatial abilities did not necessarily lead to a numerical representation impairment, the collection comparison performance being similar in both groups, revealing however a deficit in visuo-spatial skills.

### Impact of the presentation mode on performance: Sequential vs. simultaneous

Our results demonstrated a specific deficit in discrete numerical processing for non-symbolic stimuli which was observed only when stimuli were presented sequentially and this whatever the modality of presentation. These results cannot be accounted for by the use of counting in these sequential presentations. Indeed, the stimuli in the sequential mode were presented for a very short duration (varying from 66.7 to 300 ms) preventing any counting process from taking place (the counting speed by item was 566 ms on average).

Our data are in line with those of Silbert, Wolff [39] who demonstrated a specific deficit in temporal/serial processing in women with TS. Indeed, they pointed out that TS participants scored significantly lower in comparing, memorizing and transforming several sequences of sounds (rhythmic pattern, pairs of tonal sequences and a famous melody) in comparison to control participants. Altogether, these results suggest that the sequential presentation mode would require specific processes different to those involved in a simultaneous presentation in order to construct a representation of number magnitude.

Some studies support this distinction between simultaneous and sequential numerical processing by showing distinct behavioral profiles and neural networks associated with these two presentation modes in humans and in monkeys [59, 64–66]. Tokita and Ishiguchi [66] measured the Weber fraction in a numerosity comparison task contrasting sequential, simultaneous, and cross-mode stimuli in young adults. Despite great individual differences, performance differed between the simultaneous and the sequential conditions and was systematically worse in the cross-format condition than in the simultaneous condition. In the same way, Benoit, Lehalle [67] observed in young children better abilities to compare simultaneous numerosities than sequentially presented ones. Using fMRI in adults, Dormal, Andres [59] showed that some brain areas such as the central part of the right intraparietal sulcus were activated for both simultaneous or sequential presentations but that there was also specific brain activity for numerosity extraction in each of the presentation modes. Indeed, simultaneous presentation independently activated a network of left posterior parietal areas, probably linked to the visuo-spatial complexity of the task (see also [68, 69]) while sequential presentation involved a right-lateralized activity including the inferior parietal and frontal gyrus and supplementary motor area, areas also implicated in STM processing. The authors suggested that this right activation might reflect the involvement of a specific system allowing the serial position of each item in a sequence to be followed.

Some authors do not share this view [8, 20]. For instance, Barth, La Mont [20] claim that preschool children, like adults, possess approximate number representations that are independent of the modality or format of the stimuli to be enumerated. In support of this, they showed that preschoolers were able to compare the numerosity of sequences of tones and dot arrays.

The present results clearly show that the presentation mode of the stimuli are of significant importance in measuring the acuity of number magnitude. However, two different hypotheses may be considered in the face of these results. First, these results could be interpreted as arguing against the hypothesis of a single abstract numerical magnitude representation [8, 20] and support the view of magnitude representations depending upon the presentation mode of the stimuli, for instance, a sequential magnitude representation and a simultaneous magnitude representation. Our results indeed argue against the hypothesis of an amodal numerical representation since we observed a deficit only when numerical stimuli were presented sequentially, meaning that the modality of presentation directly influenced performance.

An alternative interpretation is to consider that there is a unique approximate number magnitude representation but that in order to develop this representation, different pre-numerical processes would come into play according to the presentation mode of the stimuli. Basically, in a simultaneous condition, numerosity is extracted by accumulating the elements displayed in different spatial locations (as in the topographic map [52]) while in the sequential mode, numerosity is extracted from the accumulation of events distributed in time. Accordingly, measuring number acuity is necessarily confounded with measuring the functioning of these pre-numerical accumulating processes.

Currently, it is still unclear what processing operates in order to extract numerosities with a sequential presentation, even if some theories may be put forward. When the elements of a set are presented sequentially, participants have to process successive events occurring one at a time and then accumulate those events to determine the global magnitude of the set. In the accumulator model, Gelman and Gallistel [70] proposed three distinct and successive steps, namely, the accumulation process in an internal counter proceeding by incrementation of the successive items, the temporary retention in memory and finally the decision-making in the case of a comparison task. In the present study, the decision-making process cannot explain the sequential deficit since if this was the case, other comparison tasks should be impaired as well. In contrast, the two other steps, such as the accumulation process and the temporary retention in memory, could explain a significant part of the difference observed between the two groups, since both processes require STM abilities. In this study, we observed that the TS group presented lower STM capacity than the C group and that controlling for STM abilities completely erased the group differences in sequential numerical comparison tasks.

### Potential mediation of STM abilities in the relation between magnitude representation and mathematical abilities

A final related concern is the question of the link between the deficit in sequential numerical processing and the arithmetical difficulties evidenced in TS. Our data did not demonstrate a strong relationship between magnitude abilities and mathematical performance. Thus, people with TS presented at the same time a deficit in mathematical abilities and in processing sequential numerical information but only the numerical processing of sound sequences correlated with arithmetical performance (no correlation with the dot sequence comparison abilities). This is in line with a recent review highlighting the unclear association between non-symbolic abilities and mathematical performance in healthy and dyscalculic children and adults [71]. Such an association may have been observed earlier in TS development but our population, mainly composed of adults, would have largely compensated for their difficulties during development. A more likely explanation supported by our data is that the deficits in sequential numerical information processing and in arithmetic are mediated by a third factor making an indirect link between those deficits. Our results suggest that STM is a good candidate to explain both deficits. Indeed, when controlling for memory capacities, the correlation between the sounds sequence and arithmetical capacities became completely non-significant. Globally, STM measures were the stronger predictors of mathematical achievement in our study in accordance with a large body of the literature which has underlined the key role played by STM in mathematical abilities (see [11] for a review). However, it is important to note that Turner syndrome cannot be a profile relevant for all mathematical learning disabilities. This syndrome was a unique opportunity to study the association between magnitude representation, visuo-spatial and STM abilities but not all children with mathematical disabilities exhibit this association of difficulties.

Before concluding this research it is worth considering the characteristics of the sample used here. Indeed, due to the fact that we were testing participants of a genetic disorder, it was impossible to obtain a sufficiently large sample of people with about the same age. As a consequence, we tested children, teenagers and adults. One should thus consider the possibility that the profile of results obtained here might change with development. In order to address this question but retaining similar power in the analyses, we ran new ANCOVAs on all tasks but adding a supplementary variable, i.e., the age group. In a first series of analyses we split the group around the age of 12 (6 pairs of children and 6 pairs of teenagers of adults) and in another series of analyses, we split the group around the age of 18 (9 pairs of children or teenagers and 11 pairs of adults). In none of the cases did the age group interact with the task or with the task and group (TS or C). Accordingly, it seems that the profile observed here does not significantly change with development. However, future research with larger samples of different ages might consider studying this question more carefully.

### Conclusion

This study challenges a number of assertions in the current literature. First, our use of an appropriate methodology has supplied data permitting us to confirm that basic number deficit in TS is not influenced by TS participants’ lower spatial skills—indeed, their collection and length comparison abilities are intact—but seems rather to be prompted by their STM and WM deficit due to failure in apprehending the sequential presentation of stimuli. Second, these results supply better knowledge of TS abilities in more basic numerical processing. Third, these data in TS represent the first neuropsychological evidence of distinct coding processes for simultaneous and sequential numerosity extraction independently of modality and perceptual variables. Our results add to the accumulating evidence that fails to corroborate the abstract nature of magnitude representation and are in line with animal and human neuroimaging data and computational models suggesting distinct representations for number magnitude according to the presentation mode or distinct pre-numerical processes in elaborating number magnitude representation. More generally, contrasting numerical comparison tasks relying on different modalities and presentation modes underline the importance of considering simultaneous and sequential presentation when we assess non-symbolic magnitude representation, simultaneous presentation often being preferred in the literature. Future studies should examine more precisely the developmental association between specific abilities in each presentation format and later mathematical abilities.

## Supporting information

S1 Data General information and general measures for both groups (VSSP = Visuo spatial sketchpad; PL = Phonological loop; CE = central executive). (XLSX)

S2 Data Weber fractions for non symbolic magnitude comparison tasks (NA = Arabic numbers; NVO = Verbal numbers). (XLSX)

S3 Data Median correct response times in function of ratios for symbolic magnitude processing tasks (NA = Arabic numbers; NVO = Verbal numbers). (XLSX)

S4 Data Raw data for the subitizing task (ACC = Accuracy; RT = Response Time). (XLSX)
